# Drug-resistant tuberculosis profiles among patients presenting at the antituberculosis center of Brazzaville, Republic of Congo

**DOI:** 10.1186/s12941-025-00786-8

**Published:** 2025-05-09

**Authors:** Breli Bonheur Ngouama, Jean Claude Djontu, Darrel Ornelle Elion Assiana, Freisnel Hermeland Mouzinga, Mita Naomie Merveille Dello, Jabar Babatunde Pacome Agbo Achimi Abdul, Christopher Mebiame Biyogho, Rhett Chester Mevyann, Guy Arnault Rogue Mfoumbi Ibinda, Micheska Epola Dibamba Ndanga, Franck Hardain Okemba Okombi, Michel Illoye Ayet, Lemercier Khunell Siele, Roélie Foxie Mizele Kitoti, Jeannhey Christevy Vouvoungui, Alain Maxime Mouanga, Alain Brice Vouidibio Mbozo, Veronique Penlap, Ayola Akim Adegnika, Martin Peter Grobusch, Timothy D. McHugh, Ali Zumla, Francine Ntoumi

**Affiliations:** 1 Fondation Congolaise pour la Recherche Médicale, Brazzaville, Republic of the Congo; 2 Faculté des Sciences et Techniques, Université Marien Ngouabi, Brazzaville, Republic of the Congo; 3 Centre Antituberculeux de Brazzaville, Brazzaville, Republic of the Congo; 4 Programme National de Lutte contre la Tuberculose, Brazzaville, Republic of the Congo; 5 Centre de Recherches Médicales de Lambaréné, Lambaréné, Gabon; 6 Institute of Tropical Medicine, University of Tübingen, Tübingen, Germany; 7 Center for Tropical Medicine and Travel Medicine, Amsterdam, The Netherlands; 8 Centre for Clinical Microbiology, Division of Infection and Immunity, University College London, London, UK; 9 NIHR Biomedical Research Centre, UCL Hospitals NHS Foundation Trust, London, UK; 10 Faculté des Sciences de la Santé, Université Marien Ngouabi, Brazzaville, Republic of the Congo; 11 University Yaoundé1, Yaoundé, Cameroon; 12 German Center for Infection Research (DZIF), Partner site Tübingen, Tübingen, Germany

**Keywords:** Drug resistant *Mycobacterium tuberculosis*, Xpert MTB / RIF, MTB/XDR, Republic of Congo

## Abstract

**Background:**

WHO strategy to end Tuberculosis (TB) calls for drug susceptibility testing of *Mycobacterium tuberculosis* (MTB) for all patients, in high TB burden settings. Thus, this study aimed to investigate the MTB drug resistance profiles and related risk factors among patients presenting to the Antituberculosis Center of Brazzaville, Republic of Congo.

**Methods:**

A cross-sectional study was carried out from July 2022 to August 2023 involving 1,121 presumptive pulmonary tuberculosis patients enrolled to the Antituberculosis Center of Brazzaville. Sputum samples were collected from all the study participants for the diagnosis of tuberculosis and rifampicin resistance, using the Xpert MTB/RIF (Cepheid, USA) assay. Samples positive for MTB with drug resistance to RIF were further tested for the second line anti-MTB drug susceptibility using the 10-color Xpert MTB/XDR assay.

**Result:**

Out of 1,121 presumptive TB patients tested, 302/1,121 (26.9%) were MTB positive. Among these, 18/302 (6.0%) had received previous TB treatment and 15/302 (5.0%) were HIV co-infected. The mean age of the study population was 34 years, with a higher prevalence in males (69.2%). Of the MTB isolates, 25/302 (8.3%) were Rifampicin-resistant, with 24/25 (96%) further confirmed as multi-resistant strains, including 6/24 (25%) pre-XDR. Risk factors for MDR-TB included a history of TB treatment (AOR = 8.96, *p* = 0.002) and chronic cough (AOR = 7.14, *p* = 0.003).

**Conclusions:**

This study reveals a high level of drug-resistant tuberculosis in Brazzaville, with previous TB treatment being a significant risk factor. The findings underscore the need to strengthen molecular surveillance and TB management and control measures in the Republic of Congo.

## Introduction

Tuberculosis (TB) is a major global health issue caused primarily by *Mycobacterium tuberculosis*. Despite effective treatment, TB remains a significant health threat. In 2023, there were approximately 10.8 million new TB cases worldwide and 1.25 million deaths, with a notable burden in high TB incidence countries. The WHO’s End TB Strategy aims to reduce TB incidence and mortality through various measures, including rapid detection of drug-resistant strains. Multidrug-resistant TB (MDR-TB) and extensively drug-resistant TB (XDR-TB) are particularly challenging, as they require more complex treatment regimens and pose greater public health risks [1].].

World Health Organization (WHO) and United Nation (UN) Member States committed to ending the TB epidemic through the adoption of WHO’s End TB Strategy. The target of this strategy included the annual decline in the TB incidence rate of 4–5% per year by 2020, accelerating to 10% per year by 2025 and then to an average of 17% per year from 2025 to 2035 [2, 3]. Clearly, the emergence and spread of drug resistant tuberculosis represents a serious challenge for the achievement of this goal in many countries [4, 5] Multidrug resistant tuberculosis (MDR-TB), is defined as resistance to isoniazid and rifampicin, the backbone of standard therapy while XDR-TB is defined as a TB caused by *Mycobacterium tuberculosis* strains that fulfil the definition of MDR/RR-TB and that are also resistant to any fluoroquinolone and at least one additional Group A drug (levofloxacin or moxifloxacin, bedaquiline and linezolid) [6, 7] In high TB burden countries, WHO recommends the rapid detection of Rifampicin resistance (RR) strains, which is used as a proxy for MDR-TB [1].

Globally, an estimated 400 000 people developed multidrug-resistant or rifampicin-resistant TB (MDR/RR-TB) in 2022, with 60,000 MDR/RR-TB cases being reported in Africa [1], where the highest proportion of TB/HIV co-infection is also reported [1, 8]. The most difficult and complicated form of drug resistant TB is known as extensively drug resistant tuberculosis (XDR-TB) reported from several countries, including the Republic of Congo [9].

The Republic of Congo is one of thirty TB high burden countries. In 2023, around 14,370 TB cases were notified in the country, which has an estimated population of 6.14 million (Ref). The overall TB incidence rate was 369 cases per 100,000 inhabitants in the general population and 112 cases per 100,000 inhabitants among individuals with HIV [10] The TB related mortality rate was estimated at 46 per 100, 000 inhabitants for HIV-negative individuals and 39 per 100,000 inhabitants for HIV-positive individuals respectively. However, as mentioned in the WHO TB reports for 2021,2022 and 2023, there were no national drug resistance survey or surveillance in the Republic of Congo, therefore there is limited data at hand on the prevalence and distribution of MDR-TB across the country [11, 12].

According to the TB national control program, MDR/RR-TB accounted for 5.1% of cases in the Republic of Congo in 2022, with only 37 cases of pre-XDR and two cases XDR being detected across the country [10]. This may probably reflect an underestimation of cases due to limited and insufficient testing capacity, rather than a low prevalence of MDR/RR TB in the country, as testing coverage was only 31%, with GeneXpert machines installed in just 5 out of the 12 departments [10, 13, 14].

To feel this gap, a total of 23 GeneXpert (MTB) platforms were installed in 2023 in the 11/12 Departments of the Republic of Congo among which 11 (10-color Xpert MTB/XDR Xpert) used for XDR-TB resistance testing and 12 (6-color Xpert MTB/RIF), for RR-TB testing. These machines replaced AFB smear and microscopy for the initial diagnosis of all presumptive TB patients at centers equipped with Xpert, as well as the evaluation of presumptive drug resistance in samples from patients in areas without the machine Xpert (a sample transportation system is in place for these specific patients). However, despite these logistic efforts, the national coverage of MTB drug resistance testing remains low (40%) [10].

Apart from the above mentioned TB report, which are typically based on administrative records that may have limitations in consistency, accuracy, and completeness, the last study on TB drug resistance in the Republic of Congo was conducted in 2017.This study reporteda 9.8% prevalence of MDR/RR-TB in patients with no history of TB treatment [15]. Although The study faces limitation (with small samples size: 92 participants) and being conducted in peripheral screening and treatment centers (CDTs), the obtained prevalence of MDR/RR-TB was significantly higher compared to the data from the TB national control Program (5.1%). While this may not be directly comparable, it underscores the potential underestimation of MDR-TB in the country.Given these challenges, our study sought to fill this knowledge gap by investigating the drug resistance profiles of confirmed TB patients in Brazzaville, the capital and largest city of the Republic of Congo. We focused on the prevalence of MDR and XDR-TB, as well as the risk factors associated with drug-resistant TB in this setting.

## Methods

### Study site

The study was conducted, and participants enrolled, at the Antituberculosis Center of Brazzaville, which receives samples from peripheral TB screening and treatment centers across the city. Brazzaville is the political and administrative capital of the Republic of Congo and hosts 35% (2 145 783) of the overall country population.

### Study design and population

A cross-sectional study was conducted between 16th July 2022 and 02nd August 2023 to estimate the prevalence of drug resistant (first line anti-TB drugs, MDR-TB or XDR-TB). The target population included patients with at least eight years of age, with symptoms of a history of cough for more than two weeks or pulmonary X-Ray abnormalities, newly presumptive or previously treated for TB. Additional data including TB treatment history, contacts, socio demographic and clinical data were collected using a standard well-structured questionnaire.

### Definitions

#### Presumpitve tuberculosis participants

Individuals with a history of TB treatment or not with symptoms of TB (coughing for 2 weeks or more, persistent and productive, sputum).

#### Chronic cough

Cough lasting more than eight weeks.

#### Persisting cough

History of cough for more than two weeks.

#### MDR

TB with resistance to at least both Isoniazid and Rifampicin.

#### RR

TB with resistance to Rifampicin.

#### PreXDR

TB MDR and resistant to a fluoroquinolone.

#### XDR

TB MDR and also resistant to any fluoroquinolone and at least one additional group A drug (levofloxacin, moxifloxacin, bedaquiline, and linezolid).

### Ethical considerations

Approval was obtained from the ethics committee of the *Fondation Congolaise pour la Recherche Médicale*. Prior to enrolment, participants were informed about the purpose and the protocol of the study. Written informed consent was obtained from all individuals willing to participate to this study. For participants between 8 and 17 years old, assent was obtained from the individuals and signed informed consent was obtained from parents or guardians.

### Samples collection and treatment

Early morning sputum samples were collected from all enrolled participants, liquefied and processed by Xpert MTB/RIF ultra to simultaneously detect the presence of *M. tuberculosis* and RIF resistance as per the manufacturers instructions. For patients with MTB infection and rifampicin resistance, additional sputum samples were collected the next day, which corresponded to the day the results were retrieved. These samples were then transported to the Centre de Recherches Médicales de Lambaréné (CERMEL), where they were subjected to the 10-color Xpert MTB/XDR assay for the assessment of second-line drug resistance. The Xpert MTB/XDR assay is a 9-plex assay consisting of 10 sloppy molecular beacon probes that target eight different M. tuberculosis genes mutations involved in MTB resistance to isoniazid (INH); fluoroquinolones (FLQ); ethionamide (ETH); Amikacin, Capreomycin and Kanamycin [16]. For all individuals with at least GeneXpert MTB detected result, blood samples were collected for HIV test using the Determine HIV 1/2 rapid test (Alere GmbH, Cologne, Germany), and the Bioline (Abbott Diagnostics Korea Inc3) and Unigold (Trinity Biotech plc, Wicklow, Ireland) rapid tests in accordance with the national guidelines.

### Data analysis

The data were analyzed by using Graph Pad Prism (Version 8.0.2). The results were presented using descriptive statistics, and frequency distributions of the variables were computed using tables. Multivariate analyses were performed for categorical variables and odds ratios were used to measure the strength of the association between potential risk factors and drug resistance TB. *P*-value < 0.05 was considered significant.

## Results

### General characteristics of the study population

**Fig. 1:**
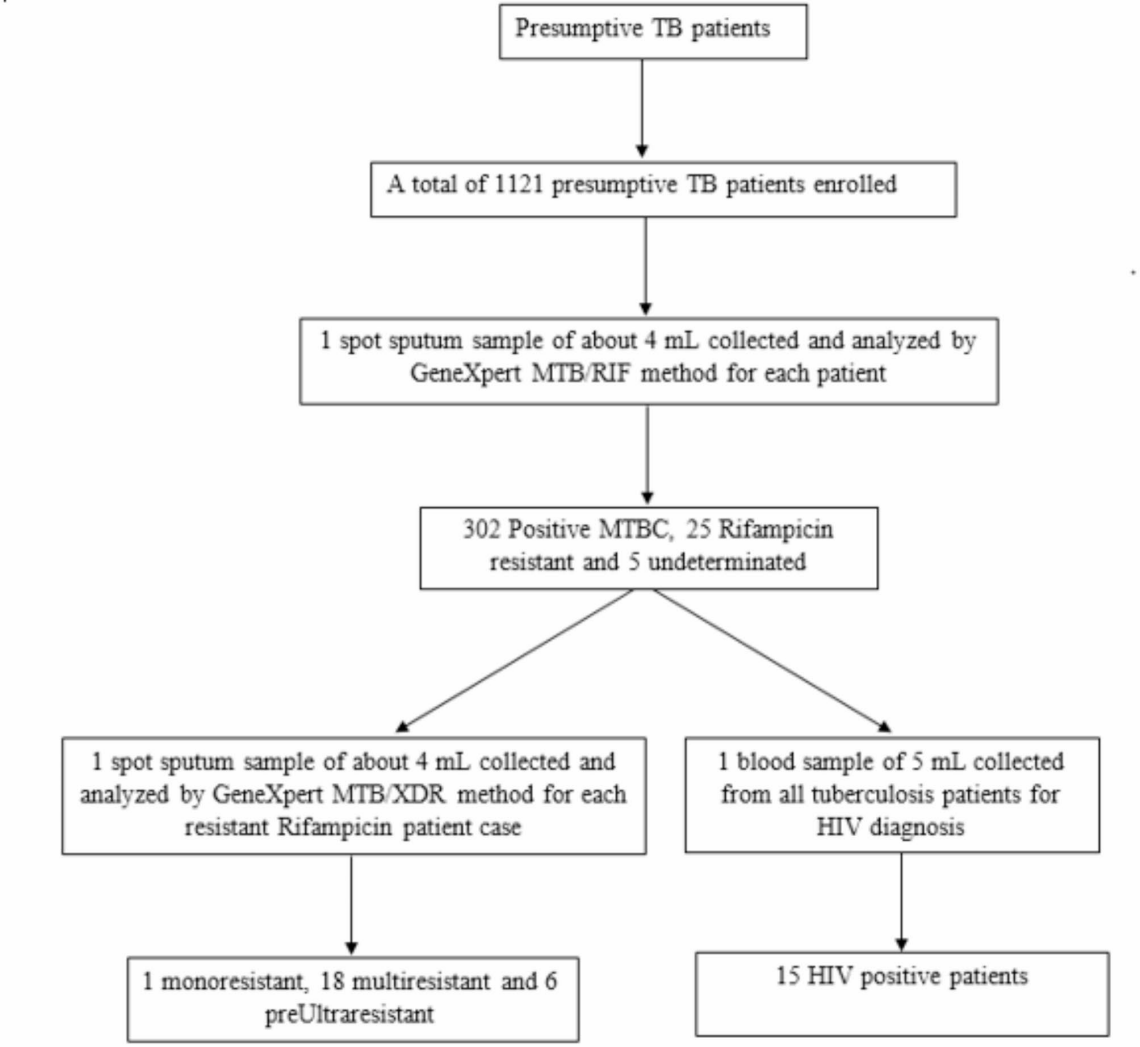
Flowchart of patient recruitment at the antituberculosis Center of Brazzaville and specimen analysis

Figure 1; Table 1 depicts the characteristics of the study population and their enrolment. From 16th July 2022 to 02nd August 2023, a total of 1,121 presumptive TB patients residing in the nine districts of Brazzaville were enrolled in this study. 26.9% (302/1,121) were confirmed positive for TB by GeneXpert including 69.2% (209/302) from males and 30.8% (63/302) from females. The age of participants ranged from 8e to 80 years, with a mean age of 34 years. Newly diagnosed TB positive patients represented 94% (284/302) of study participants while 6% (18/302) had a history of TB treatment. Considering personal behavior, 29.8% (90/302) patients were smokers (cigarette consumers) and 57.6% (174/302) regular alcohol consumers; 13.9% (42/302) were unemployed. 32.1% (97/302) of TB confirmed patients had a chronic cough, 97.4% (294/302) had a fever and 80.8% (244/302) had lost weight. A total of 15/302 (5%) of the overall TB patients were HIV co-infected.

**Table 1:** Socio-demographic, clinical, and behavioral characteristics of TB patients (*n* = 302)

Variables	Tuberculosis Patients (*n* = 302)	Proportion (%)
Age in years		
[8–21)	41	13.6
[21–41)	183	60.6
[41–61)	62	20.5
≥ 61	16	5.3
Gender		
Female	93	30.8
Male	209	69.2
Study Level		
No formal education	6	2.0
Primary school	49	16.2
Secondary school	204	67.5
Higher education	43	14.2
History of TB Treatment	18	6.0
Smoking	90	29.8
Alcohol Abuse	174	57.6
HIV Status		
Yes	15	5.0
No	287	95.0
Chronic Cough	97	32.1
Fever	294	97.4
Weight Loss	244	80.8
Occupation		
Employed	260	86.1
Unemployed	42	13.9

### Drug resistant MTB strains

Among the 302 MTB confirmed patients, the prevalence of drug resistant MTB (DR-TB) was 25/302 (8.3%); with 19/24 (79.2%) being newly diagnosed, and 5/24 (20.8%) patients with a history of TB treatment. Among the drug resistant TB patients, 24/25 (96%) were resistant to both isoniazid and Rifampicin (MDR), 1/25 (4%) was exclusively resistant to rifampicin (RR) and 6/25 (24%) had pre-XDR strains (four for fluoroquinolone and two for injectable second line drugs).

### Factors associated with MTB drug resistance

Table 2 shows results for multivariate analysis of the association between RR-TB status among tuberculosis patients and socio-demographic, clinical and behavioral factors. DR-TB was significantly associated with history of TB (OR = 3.99, *p* = 0.002) and chronic cough (OR = 4.51, *p* = 0.003) while HIV infection, smoking, alcohol abuse, sex, age, fever, weight loss, study level and occupation were not associated to DR-TB in this cohort.

**Table 2:** Multivariate analysis of the association between RR-TB status among tuberculosis patients and socio-demographic,** clinical and behavioral factors.**

Variables	*N*	TB Resistant patients *n* (%)	Crude Odd Ratio (CI.95%)	Adjusted Odd Ratio (CI.95%)	*P*. value
Age in years					
[8–18[	21	2 (9.52)	1	1	
[18–44[	224	17 (7.59)	0.78 (0.17–3.63)	0.31 (0.04–2.59)	0.283
≥ 45	57	4 (7.02)	0.72 (0.12–4.24)	0. 30 (0.02–3.09)	0.311
Sex					
Female	93	6 (6.45)	1	1	
Male	209	17 (8.13)	1.28 (0.50–3.36)	2.02 (0.01–2.69)	0.208
Study level					
Primary school	55	7 (12.73)	1	1	
Secondary	204	13 (6,37)	0.47 (0.18–1.23)	0.37 (0.12–1.12)	0.078
Higher	43	3 (7.62)	0.51(0.12–2.11)	0.56 (0,11 -2.82)	0.487
History of TB treatment					
Yes	18	5 (27.78)	5.68 (1.82–17.71)	8.96 (2.37–33.94)	0.002
No	284	19 (6.69)	1	1	
Smoking					
Yes	90	5 (5.56)	0.63 (0.25–1.72)	0.39 (0.12–1.32)	0.131
No	212	18 (8.49)	1	1	
Alcohol abuse					
Yes	174	15 (8.62)	1.41 (0.58–3.45)	2.36 (0.77–7.24)	0.207
No	128	8 (6.25)	1	1	
HIV status					
Positive	15	2 (13.33)	1.95 (0.41–8.69)	2.32 (0.41–13.25)	0.534
Negative	287	21 (7.32)	1	1	
Chronic cough					
Yes	146	18 (12.33)	4.25 (1.53–11.76)	7.14 (1.95–26.08)	0.003
No	156	5 (3.21)	1	1	
Fever					
Yes	294	21 (25.00)	3.50 (0.71–16.15)	0.29 (0.06–1.42)	0.152
No	8	2 (7.14)	1	1	
Weight loss					
Yes	244	20 (8.20)	1.63 (0.51–5.36)	1.98 (0.51–7.71)	0.323
No	58	3 (5.17)	1	1	
Occupation					
Employed	106	7 (6.60)	0.79 (0.32–1.99)	0.56 (0.03–12.38)	0.429
Unemployed	196	16 (8.16)	1	1	

## Discussion

This health facility-based cross-sectional survey was designed to assess the drug resistance profile of *Mycobacterium tuberculosis* (MTB) isolates and identify associated risk factors among patients at the Antituberculosis Center of Brazzaville. The study screened 1,121 individuals with presumptive tuberculosis, of whom 302 (26.9%) tested positive for pulmonary tuberculosis. This highlights the critical importance of confirming all presumptive TB cases with recommended diagnostic methods before initiating treatment.

The overall prevalence of drug resistance in this study was 8.3%. This included 6.7% in newly diagnosed patients and 27.8% in those with a history of TB treatment. Notably, within the resistant cases, 4% were exclusively resistant to Rifampicin (RR-DR), 72% were multidrug-resistant (MDR), and 24% were pre-extensively drug-resistant (Pre-XDR). The prevalence of DR-TB observed in this study is significantly higher compared to the 2.7% reported by Okemba-Okombi and colleagues in a 2017 study conducted at the same center, albeit with a smaller sample size (*n* = 88) [17]. Additionally, the 8.3% prevalence of DR-MTB found in our study exceeds the 5% national prevalence reported in 2022. This discrepancy may be attributed to the higher capacity for DR-TB testing in Brazzaville compared to other regions of the Republic of Congo, where many drug-resistant cases may remain undetected.

Our findings insignificantly contrast with the 9.8% prevalence of drug resistance in patients with no history of TB treatment we reported in another health facility in southern Brazzaville [15]. This slight lower prevalence might reflect a decrease in DR-TB transmission in Brazzaville in recent years with the implementation of GeneXpert as the initial diagnostic test for tuberculosis in November 2019 for site equipped with the tool. However, the smaller sample size and more limited geographical scope of previous study could also account for the differences observed.

When compared to recent data from other African countries [18–20], the high prevalence of Pre-XDR/XDR strains in our study is concerning. This finding suggests that without intensified control measures, the situation could deteriorate further. For instance, the prevalence of DR-TB among previously treated patients in our study (22%) is higher than reported in some high-burden countries. For example, studies from Vietnam and the Democratic Republic of Congo have reported MDR/RR-TB prevalence rates of 1.7% and 5%, respectively, in new cases, and up to 9.5% and 20% in previously treated patients [21–23].In South Africa, the prevalence was 3.4% among new cases and 7.1% among previously treated patients in 2019 [24].

Our multivariate analysis identified a history of TB treatment (*p* = 0.002) and chronic cough (*p* = 0.003) as significant risk factors for drug-resistant tuberculosis. These findings are consistent with previous studies which have reported increased incidence of drug resistance among individuals with prior TB treatment [23, 25–29]. Previous TB treatment as well as previous infection with MTB may leave residual damage in the pulmonary tract, which can create a conducive environment for new MTB strains to acquire drug resistance [30]. Additionally, resistant strains are known to be more virulent, which may explain the association between chronic cough and increased risk of drug resistance observed in this study. As the infection progresses, the tissues of the lungs become damaged, leading to a continuous cough [31, 32].

Conversely, no significant associations were found between drug resistance and factors such as HIV infection, smoking, alcohol abuse, sex, age, fever, weight loss, diarrhea, TB contact, or occupation. The low prevalence of HIV/TB co-infection in our study (5%) contrasts with higher rates reported elsewhere [23–28]. This could be attributed to the success of recent preventive TB treatments among HIV/AIDS patients in the Republic of Congo, including an increase in the proportion of HIV patients receiving cotrimoxazole preventive therapy—from 36% in 2016 to 78% in 2023 [10, 33].

Our study had several limitations. One notable limitation of this study is the absence of drug susceptibility testing (DST) for Pyrazinamide (PZA) and Ethambutol (ETH), due to the constraints of the Xpert MTB/XDR assay. This limitation means that the full resistance profile of MTB could not be assessed, potentially underestimating the extent of drug resistance.

## Conclusion

The study reveals a concerningly high prevalence of drug-resistant tuberculosis in Brazzaville, with a notable emergence of XDR strains. The significant association of drug resistance with a history of TB treatment and chronic cough underscores the need for improved management strategies and diagnostic capacities as well as enhanced surveillance.

## Data Availability

Raw data will be made available upon request to the corresponding author.

## Abbreviations

MDR-TB: Multidrug- Resistant-Tuberculosis
TB: Tuberculosis
XDR-TB: Extensively drug-resistant-Tuberculosis
RR-TB: Rifampicin-resistant-Tuberculosis
WHO: World Health Organization
RR-TB: Rifampicin-resistant tuberculosis
MTB: *Mycobacterium tuberculosis;*
CDTs: TB screening and treatment centers
